# Comparative analysis of fecal microbial communities in cattle and Bactrian camels

**DOI:** 10.1371/journal.pone.0173062

**Published:** 2017-03-16

**Authors:** Liang Ming, Li Yi, Siriguleng, Surong Hasi, Jing He, Le Hai, Zhaoxia Wang, Fucheng Guo, Xiangyu Qiao, Jirimutu

**Affiliations:** 1 Key Laboratory of Dairy Biotechnology and Bioengineering, Ministry of Education, College of Food Science and Engineering, Inner Mongolia Agricultural University, Hohhot, Inner Mongolia, China; 2 Camel Research Institute of Inner Mongolia, Alashan, Inner Mongolia, China; 3 College of Veterinary Medicine, Inner Mongolia Agricultural University, Hohhot, Inner Mongolia, China; Universidade de Aveiro, PORTUGAL

## Abstract

Bactrian camels may have a unique gastrointestinal (GI) microbiome because of their distinctive digestive systems, unique eating habits and extreme living conditions. However, understanding of the microbial communities in the Bactrian camel GI tract is still limited. In this study, microbial communities were investigated by comparative analyses of 16S rRNA hypervariable region V4 sequences of fecal bacteria sampled from 94 animals in four population groups: Inner Mongolian cattle (IMG-Cattle), Inner Mongolian domestic Bactrian camels (IMG-DBC), Mongolian domestic Bactrian camels (MG-DBC), and Mongolian wild Bactrian camels (MG-WBC). A total of 2,097,985 high-quality reads were obtained and yielded 471,767,607 bases of sequence. Firmicutes was the predominant phylum in the population groups IMG-Cattle, IMG-DBC and MG-WBC, followed (except in the Inner Mongolian cattle) by Verrucomicrobia. Bacteroidetes were abundant in the IMG-DBC and MG-WBC populations. Hierarchical clustered heatmap analysis revealed that the microbial community composition within the three Bactrian camel groups was relatively similar, and somewhat distinct from that in the cattle. A similar result was determined by principal component analysis, in which the camels grouped together. We also found several species-specific differences in microbial communities at the genus level: for example, *Desulfovibrio* was abundant in the IMG-DBC and MG-WBC groups; *Pseudomonas* was abundant in the IMG-Cattle group; and *Fibrobacter*, *Coprobacillus*, and *Paludibacter* were scarce in the MG-WBC group. Such differences may be related to different eating habits and living conditions of the cattle and the various camel populations.

## Introduction

The gastrointestinal (GI) microbiome of ruminants is a complex, dynamic ecosystem, closely related to nutrition, metabolism and immunity of the host animal. The ecology, physiological characteristics, colony structure and bacterial diversity of the gut microbiota of ruminants has been the subject of much research[1–5]. In this study, we collected fecal samples from cattle and camels to research their microbial communities.

The domestic Bactrian camel (*Camelus bactrianus*) plays numerous socioeconomic roles in the lives of millions of people in semi-dry and arid regions; these camels are used for draught power, transport, wool production, milk and meat[6]. The extant wild Bactrian camel (*Camelus ferus*) is an endangered large mammal and mainly distributed in northwest China and the Gobi Altai desert in Mongolia[7]. Domestic and wild Bactrian camels are resilient animals that can tolerate harsh climate, extremes of weather and variable temperature. Recent genetic studies have shown that the domestic Bactrian camel and the extant wild Bactrian camel have separate maternal origins, and the extant wild Bactrian camel is a distinct species with an independent evolutionary history to its domestic relative[8,9].

Cattle and Bactrian camels have different digestive systems and anatomies. The forestomach of cattle includes four chambers, the rumen, reticulum, omasum and gastric secreting abomasum, while the Bactrian camel has three chambers and no omasum[10,11]. In addition, the dietary habits and environment of Bactrian camel are different from those of cattle. Cattle mainly feed on grass, organic feed and roughage, and live on plains and grassland; Bactrian camels can eat salt-tolerant vegetation such as Chenopodiaceae, Compositae and Leguminosae, poisonous plants such as *Peganum harmala*, *Cynomorium*, and Mongolian almond, and dry, prickly and bitter plants; they can ingest virtually any kind of vegetation including shrubs and trees[12]. Domestic Bactrian camels inhabit semi-arid plains, while wild Bactrian camels prefer a habitat of arid plains and hills where plants and water are scarce.

We hypothesized that the different digestive systems, environments and eating habits of Bactrian camels and cattle would lead to distinctive intestinal microbial flora. However, there is little information available on the gastrointestinal microbiome of Bactrian camels. Here, we used MiSeq sequencing of hypervariable 16S rRNA and comparative analysis to investigate the gastrointestinal microbiomes from 94 individuals belonging to four population groups: cattle (IMG-Cattle) and domestic Bactrian camels (IMG-DBC) from Inner Mongolia, China, and domestic (MG-DBC) and wild Bactrian camels (MG-WBC) from Gobi-Altai, Mongolia.

## Materials and methods

No specific permits were required for the field studies described in this work. The owner of the land gave permission to conduct the study on this site. The cattle (IMG-Cattle) and domestic Bactrian camels (IMG-DBC) from Inner Mongolia, China, and domestic Bactrian camels (MG-DBC) from Gobi-Altai, Mongolia, are not endangered or protected species; however, the wild Bactrian camels (MG-WBC) from Gobi-Altai, Mongolia are endangered or protected species. This study was reviewed and approved by the ethics committee of the Wild Camel Protection Foundation. Fresh fecal samples were collected from 94 individual animals. We tracked the cattle and Bactrian camels until they defecated; the fecal samples were immediately collected aseptically. The fresh fecal samples were transported to the laboratory on dry ice within 24 h of collection, and then stored at −80°C until DNA extraction.

### Fecal sample collection

Fresh fecal samples were collected from 94 individual animals: Inner Mongolian cattle (IMG-Cattle, n = 14), Inner Mongolian domestic Bactrian camels (IMG-DBC, n = 65), Mongolian domestic Bactrian camels (MG-DBC, n = 3) and Mongolian wild Bactrian camels (MG-WBC, n = 12). The IMG-cattle group belonged to the Mongolian breed, which is very robust and healthy with strong bones. This breed has a distinct ability to resist diseases and is mainly used for milking in this area. The IMG-cattle occupy much of the vast steppes, or dry grasslands, in Inner Mongolia, Xilin Gol League, China. The IMG-DBC group belonged to the Sunit Bactrian camel breed, which is also distributed in Inner Mongolia, Xilin Gol League; however, they only inhabit the Gobi desert. The MG-DBC and MG-WBC groups were distributed in the Gobi-Altai region, Mongolia, and they shared the same living environment and eating habits. Wild Bactrian camels travel in a herd of 2–15 members. In the process of sampling, we found that there were three domestic Bactrian camels in the wild Bactrian camel herd, and these domestic camels had the same diet and environment as the wild camels. We hypothesized that having the same environment and eating habits may result in the same intestinal microbial biota in the Mongolian wild and domestic Bactrian camels; because we found only three individual domestic Bactrian camels we sampled these.

In this study, the four population groups grazed freely. We used sterile tubes to collect fecal samples, which were transported to the laboratory on dry ice within 24 h of collection and stored at −80°C until DNA extraction. The maximum time fecal samples were stored prior to DNA extraction was 1 month.

### Genomic DNA extraction

Microbial genomic DNA was extracted from each sample using the QIAamp DNA stool mini kit (Qiagen, Valencia, CA, USA) according to the manufacturer’s protocol. The quality and quantity of DNA were determined with a NanoDrop (ND-1000) spectrophotometer (Thermo Fisher Scientific, Waltham, MA, USA). DNA integrity was determined using 1% agarose gel electrophoresis.

### Amplification of 16S rRNA genes

The V4 hypervariable region of bacterial 16S rRNA was amplified using universal primers 520F (5ʹ-AYTGGGYDTAAAGNG-3ʹ) and 802R (5ʹ-TACNVGGGTATCTAATCC-3ʹ). The PCR conditions were: one predenaturation cycle at 94°C for 30 s, 25 cycles of denaturation at 94°C for 15 s, annealing at 50°C for 30 s, and extension at 72°C for 30 s, and one post-elongation step at 72°C for 5 min. The PCR products were purified from 2% agarose gels using the QIAquick Gel Extraction Kit (QIAGEN). Barcoded V4 amplicons were sequenced using the paired-end method on an Illumina MiSeq instrument with a seven-cycle index read.

For analysis, duplicate and low quality reads were removed from the raw data; sequences with an average phred score >25 were retained, while those with ambiguous bases (N <1), consecutive repeat bases >6 bp, primer mismatches, or sequence lengths <50 bp were removed. Sequences with an overlap >10 bp and without any mismatch were assembled using the overlapping sequence. Reads that could not be assembled were discarded. Barcodes and sequencing primers were trimmed from the assembled sequences. The raw data has been submitted to Sequence Read Archive (SAR) database, and the accessions for my submission was PRJNA344803 (SRP092077).

### Taxonomy classification and statistical analysis

We used the Ribosomal Database Project (RDP, Release 11.4)[13] to analyze taxonomy. Operational taxonomic unit (OTUs) with an identity cutoff of 97% were counted for each sample to express the richness of bacterial species. The OTU abundance of each sample was generated at the phylum and genus levels. The bacterial community indices applied here included Chao1 (richness), Shannon (diversity) and Good’s coverage using Mothur[14] with an OTU identity cutoff of 97% after implementing a pseudo-single linkage algorithm. Principal component analysis (PCA) was performed with weighted UniFrac distance[15]. The relative abundance of genera in each population group was displayed using hierarchical clustered heatmap analysis[16].

## Results

Table 1 gives detailed characteristics of the four study population groups. A total of 2,097,985 high-quality reads were obtained from the 94 fecal samples by targeting the V4 hypervariable region of bacterial 16S rRNA genes through Illumina MiSeq sequencing analysis. In total, the reads yielded 471,767,607 bases of sequence, with median sequence length 225 bp. To evaluate whether further sampling would likely yield additional taxa, rarefaction analysis was carried out for each population group (S1–S4 Figs). For all four population groups, rarefaction curves failed to reach a plateau even at the highest numbers of OTUs analyzed, implying that there was a need for further sampling and undetected OTUs in the samples. The Good’s coverage estimations were between 87.1% and 95.5% for the samples. In this study, because of the limitation of sample number (only three camels) in the MG-DBC group, we did not compare or analyze the number of OTUs, richness, diversity or bacterial community composition in this group.

**Table 1:** Detailed characteristics of four population groups in this study.

Population group^1^	Number of individuals	Geographic distribution	Habitat	Diet preference
IMG-Cattle	14	China, Inner Mongolia	Grassland	Grass, organic feed, roughage
IMG-DBC	65	China, Inner Mongolia	Semi-arid plains	The vegetation with prickly, hair, strong odor, heavy saline and alkaline, poisonous plants, shrubs, tree
MG-DBC	3	Mongolia, Gobi Altai	Arid plains and hills, scarce water sources	Poisonous plants, shrubs, trees and very little vegetation
MG-WBC	12	Mongolia, Gobi Altai	Arid plains and hills, scarce water sources	Poisonous plants, shrubs, tree and very little vegetation

^1^IMG-Cattle: cattle from Inner Mongolia; IMG-DBC: domestic Bactrian camels from Inner Mongolia; MG-DBC: domestic Bactrian camels from Mongolia; MG-WBC: wild Bactrian camels from Mongolia.

### Bacterial diversity in fecal samples from the three population groups

We found obvious variation in bacterial diversity among the IMG-Cattle, IMG-DBC, and MG-WBC groups. The total number of OTUs ranged from 24,085 (IMG-Cattle11) to 56,193 (IMG-Cattle02) in the IMG-Cattle dataset; 6,859 (IMG-DBC22) to 48,461 (IMG-DBC07) in the IMG-DBC dataset; and 13,709 (MG-WBC10) to 32,924 (MG-WBC04) in the MG-WBC dataset (S1 Table). Furthermore, we calculated the sequence diversity and richness.

The average bacterial richness was highest in the IMG-Cattle group (average Chao1: 8428), followed by the IMG-DBC group (6130) and the MG-WBC group (5929) (S1 Table). With respect to Shannon diversity, the IMG-DBC group displayed the highest value (5.90) while the MG-WBC group had the lowest (5.35).

### Bacterial community composition in feces of the three population groups

**Fig 1:**
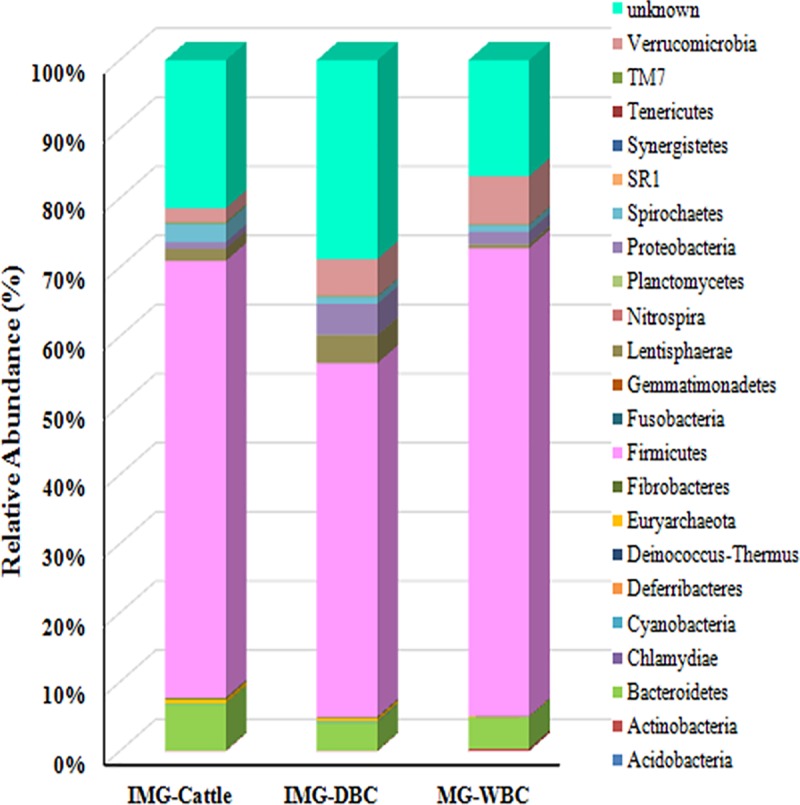
Bacterial composition in feces from different population groups (relative read abundance by phylum). Sequences that could not be classified into any known group were assigned as ‘Unknown bacteria’.

Using the Mothur program, we classified all sequences obtained from the population groups IMG-Cattle, IMG-DBC, and MG-WBC at the phylum and genus levels. We observed dissimilar 16S rRNA profiles at the phylum level across the three groups (Fig 1). The IMG-DBC group contained the highest number of phyla (21), followed by IMG-DBC (18); MG-WBC feces contained 15 phyla. In all three population groups, Firmicutes was the dominant phylum (51.19%, 67.74% and 63.23% in the IMG-DBC, MG-WBC and IMG-Cattle population groups, respectively), followed by Verrucomicrobia (5.35% and 6.97%) in the IMG-DBC and MG-WBC population groups, but followed by Bacteroidetes (6.79%) in the IMG-Cattle group. Bacteroidetes were, however, also abundant in the IMG-DBC and MG-WBC groups (4.05% and 4.52%, respectively). Phyla that were generally rarer but that were prevalent in feces of some individuals of the three population groups, were Lentisphaerae (0.47%–4.01%), Proteobacteria (0.96–4.50%) and Spirochaetes (0.97%–9.91%) (Fig 1).

In addition, we observed several phyla in the Inner Mongolian population groups (IMG-Cattle and IMG-DBC) that were not present in the Mongolian wild Bactrian camel population group, including *Deinococcus-Thermus*, *Fusobacteria*, *Gemmatimonadetes* and *Tenericutes*. At the phylum level, we also found ruminant-specific microbial communities. *Acidobacteria* was detected only in the IMG-DBC population group, *Chlamydiae* was found in the Bactrian camel population groups (IMG-DBC and MG-WBC), while *Deinococcus-Thermus*, *Fusobacteria* and *Gemmatimonadetes* were discovered in the Inner Mongolian cattle and camel population groups (IMG-Cattle and IMG-DBC). The abundance of unclassified bacteria in our study was 16.80%–28.78%.There were also differences in the genus level distribution across the three population groups. *Victivallis*, *Oscillibacter*, *Treponema*, *Blautia* and *Alistipes* displayed relatively high abundance in all three populations. *Desulfovibrio* was abundant in the two Bactrian camel groups (IMG-DBC and MG-WBC), but was scarce in the cattle group. *Pseudomonas* was abundant in the IMG-Cattle group but absent from the two Bactrian camel groups (IMG-DBC and MG-WBC). *Fibrobacter*, *Coprobacillus*, and *Paludibacter* were rare in the MG-WBC group, but were abundant in the Inner Mongolian animal groups (IMG-DBC and IMG-Cattle).

### Comparison of IMG-Cattle, IMG-DBC, MG-DBC and MG-WBC fecal microbiomes

**Fig 2:**
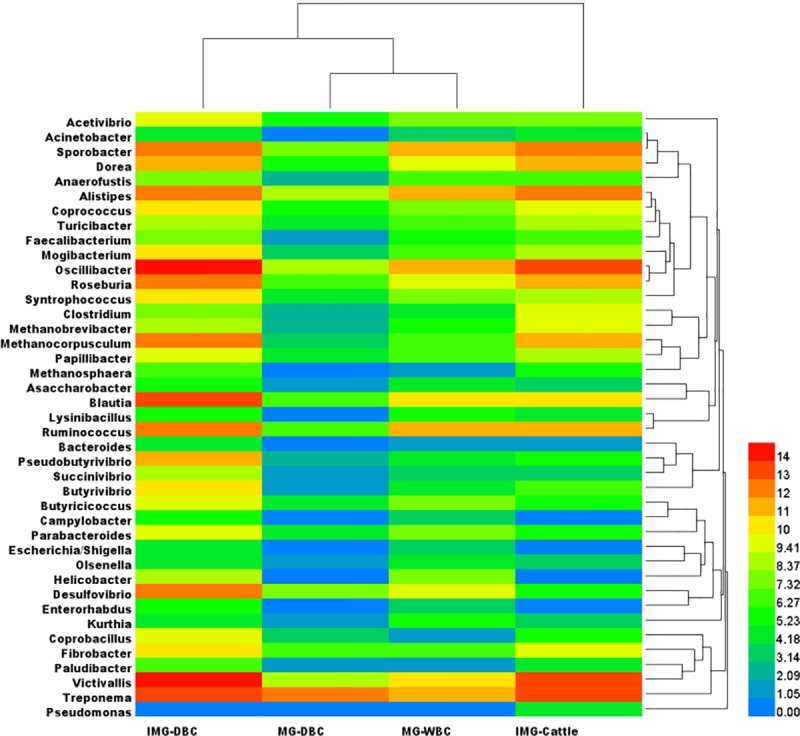
Heatmap of the relative abundance of bacterial genera in fecal samples. Blue represents the minimum relative abundance and red the maximum.

We considered relative bacterial abundance in the four population groups at the genus level (Fig 2). Hierarchical clustered heatmap analysis showed that the MG-WBC and MG-DBC groups clustered together, and then further clustered with the remaining camel group (IMG-DBC). The IMG-Cattle group formed an independent cluster.

**Fig 3:**
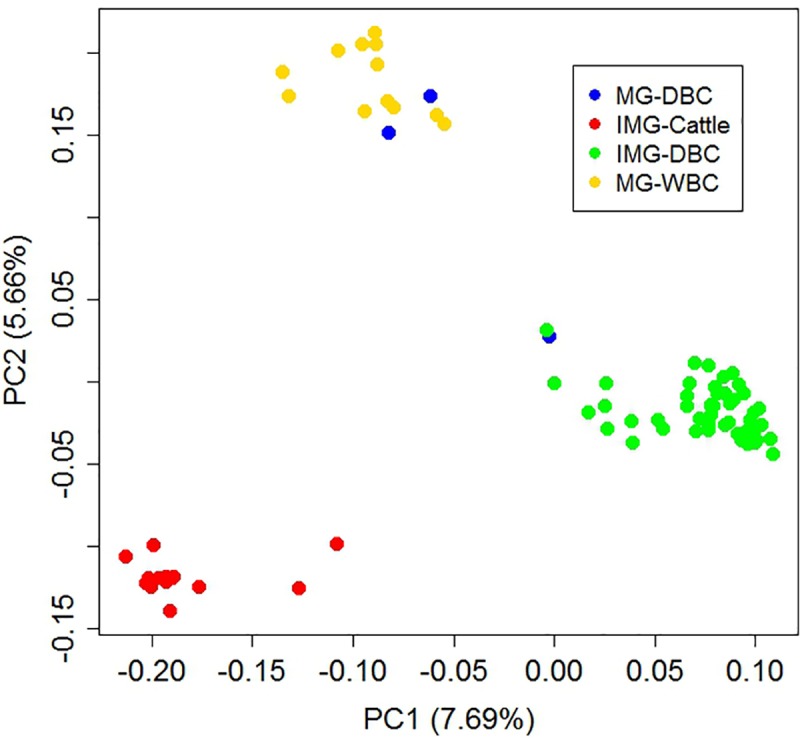
Principal component analysis (PCA) with weighted UniFrac distance analysis of the microbiomes from the four animal populations.

The principal component analysis (PCA) with weighted UniFrac distance analysis was used to compare the fecal microbiomes of the four animal populations (Fig 3). The IMG-Cattle and IMG-DBC groups inhabit the same environment in Inner Mongolia, China, but their diet is different. The MG-DBC and MG-WBC population groups come from the same environment in Gobi-Altai, Mongolia, and they have the same diet. Weighted UniFrac distance analysis of our results showed that the gut microbial communities clustered by host animal species (Fig 3); i.e., the microbiome communities from IMG-Cattle and those from the three Bactrian camel populations (MG-DBC, IMG-DBC and MG-WBC) were significantly different. Beyond that, we found that the MG-DBC population showed a greater dispersal in the PCA plot analysis than the other three population groups; one MG-DBC individual clustered with the IMG-DBC group, while two other individuals clustered with the MG-WBC population.

## Discussion

Sheep and cattle are true ruminants, whereas camels (the Bactrian camel and the dromedary camel) are described as pseudoruminants[10], which lack an omasum and only contain three stomach compartments. Several studies have been published on the microbial community of the dromedary camel[10,17–19]. In the present study, comparative analysis was undertaken of the GI microbial community in Inner Mongolian cattle, Inner Mongolian domestic Bactrian camels, Mongolian domestic Bactrian camels and Mongolian wild Bactrian camels, using a high-throughput Illumina MiSeq approach.

Because of the limited number of individuals in the Mongolian domestic Bactrian camel group (n = 3), we did not undertake analysis of the number of OTUs, richness, diversity or bacterial community composition in this group. The other three population groups (IMG-DBC, MG-WBC and IMG-Cattle) exhibited a high relative abundance of Firmicutes, which was followed by Verrucomicrobia in the camels; other phyla were relatively low in abundance.

Hierarchical clustered heatmap analysis showed that the three Bactrian camel populations clustered together while the IMG-Cattle group formed an independent cluster. This clustering may be because the three Bactrian camel populations (IMG-DBC, MG-WBC and MG-DBC) have similar digestive structures and functions that are distinct from those of the cattle. In addition, the Mongolian domestic Bactrian camel and Mongolian wild Bactrian camel grouped together initially, and then clustered with the Inner Mongolian domestic Bactrian camel. The MG-DBC and IMG-DBC populations are both domestic species and their eating habits are different. If species was the only determinant of gut microbial community composition, then the MG-DBC group and the IMG-DBC group should cluster together. However, this was not what we found: instead, different species living in the same environment (i.e. the MG-DBC and MG-WBC populations) clustered together. This implies that the environment of the host was the stronger determinant of the microbial community structure.

However, PCA with weighted UniFrac distance analysis showed that the ruminant gastrointestinal microbiomes were related to the animal host, and were perhaps species-specific or population-specific. We can relate this phenomenon to the diets of the different species. Xerophyte and halophyte vegetation forms the main part of the diet of Bactrian camels no matter where they live, and this diet is supplemented with poisonous plants such as *Peganum harmala*, *Cynomorium* and Mongolian almond. However, these plants are not eaten by cattle. So, both species and diet can explain why the microbial communities we observed in camels and cattle separated from each other. In addition, environment is a key factor in the microbial community composition, and we found that the fecal biomes of the MG-DBC and MG-WBC population groups displayed overlap.

Although the MG-DBC population sample was very limited, our result may imply significant inter-individual variability in the gut microbiome of Mongolian domestic Bactrian camels.

We observed several phyla in the Inner Mongolian population groups (IMG-Cattle and IMG-DBC) that were not present in the Mongolian wild Bactrian camel population group, suggesting that the presence of these phyla may be related to host diet and/or environment. The IMG-Cattle and IMG-DBC groups are mainly distributed in the Inner Mongolian Xilin Gol League in China; this region has an arid continental climate and native vegetation is abundant. In contrast, the MG-WBC population lives in an arid, cold climate in Mongolia (long and cold winter, short summer), with sparse vegetation. This great difference in diet and environment may result in the different gut microbial communities between the Inner Mongolian and Mongolian animal groups.

At the genus level, significant differences between different species, or between the same species in different environments, were analyzed (S2 and S3 Tables) (*p* < 0.05). We investigated the genus *Desulfovibrio*, which had fourfold or higher relative abundance (p < 0.05) in the fecal microbiome of the IMG-DBC population than in the IMG-Cattle group (S3 Table). Sulfate-reducing bacteria (SRB) are anaerobic bacteria that can produce hydrogen sulfide by reducing sulfate. *Desulfovibrio* is the predominant SRB biota among human colonic microbiota. Because hydrogen sulfide is a potential etiological agent of epithelial cells, many researchers have suggested that there is a relationship between *Desulfovibrio* and intestinal diseases[20–23]. In addition, studies showed that some *Desulfovibrio* species have bioremediation potential for toxic radionuclides[24]. Thus, the presence of so many *Desulfovibrio* in their gut may explain why Bactrian camels can survive in very harsh conditions and eat poisonous plants in a semi-arid environment. *Pseudomonas*, abundant in the IMG-Cattle, were absent from the three Bactrian camel groups. *Pseudomonas* is a bacterium with (normally) low pathogenicity but high drug-resistance. The domestic Bactrian camels contract very little infectious disease, but cattle are very different; in pasture, work to prevent epidemic infectious disease is frequently undertaken by veterinarians for each cow. This may have led to stronger drug-resistance in cattle-gut bacteria than camel-gut bacteria, which may explain the abundance of *Pseudomonas* in the cattle population samples.

Compared with the other population groups, we found that the MG-WBC group feces contained rare microbes such as *Fibrobacter* (<0.49% relative abundance), *Coprobacillus* (<0.017% relative abundance) and *Paludibacter* (<0.017% relative abundance), which may be related to the simplified diet of the wild Bactrian camel from Gobi-Altai, Mongolia, relative to the diets of the other groups. These camels inhabit an extremely harsh desert climate where their habitat ranges from rocky mountains to plains and high sand dunes. These places have scarce water sources and sparse vegetation, and the wild and domestic Bactrian camels from Gobi-Altai feed mainly on shrubs, trees and very little vegetation, which means their diet is relatively simplified.

This comparative work showed that camels from different locations and environments shared similar fecal microbial composition. For the first time, the present study applied sequencing of the 16S rRNA hypervariable region V4 of bacteria to comparative research of the fecal microbiome communities of cattle and Bactrian camels, which provides a foundation for understanding the complexity of the Bactrian camel gut microbiome. However, our research only concerned the hypervariable region V4, which may limit understanding of the microbiome community of Bactrian camels. We observed a large proportion of unclassified bacteria (i.e. with distant relationships to any known sequence in public databases). To overcome these shortcomings, additional studies such as metagenomic approaches should be carried out to gain further insight into the microbial community diversity in domestic and wild Bactrian camels.

## Supporting information

S1 Fig Rarefaction analysis of the IMG-Cattle population group. Rarefaction curves of OTUs clustered at 97% sequence identity.
(DOC)

S2 Fig Rarefaction analysis of the IMG-DBC population group. Rarefaction curves of OTUs clustered at 97% sequence identity.
(DOC)

S3 Fig Rarefaction analysis of the MG-DBC population group. Rarefaction curves of OTUs clustered at 97% sequence identity.
(DOC)

S4 Fig Rarefaction analysis of the MG-WBC population group. Rarefaction curves of OTUs clustered at 97% sequence identity.
(DOC)

S1 Table Number of OTUs and estimators of sequence diversity and richness. (DOC)

S2 Table Detailed taxonomic string for the 47 significantly different OTUs between the MG-WBC and IMG-DBC groups (classified to genus, p < 0.05. Control: IMG-DBC). (DOC)

S3 Table Detailed taxonomic string for the 48 significantly different OTUs between the IMG-DBC and IMG-Cattle groups (classified to genus, p < 0.05. Control: IMG-Cattle). (DOC)
