# Supplementary material for: Comparative analysis of fecal microbial communities in cattle and Bactrian camels
Source: PLoS One. 2017 Mar 16;12(3):e0173062. doi: 10.1371/journal.pone.0173062 (PMC5354269; doi:10.1371/journal.pone.0173062)
Supplement: S2 Fig — Rarefaction curves of OTUs clustered at 97% sequence identity. (DOC) [file pone.0173062.s002.doc]

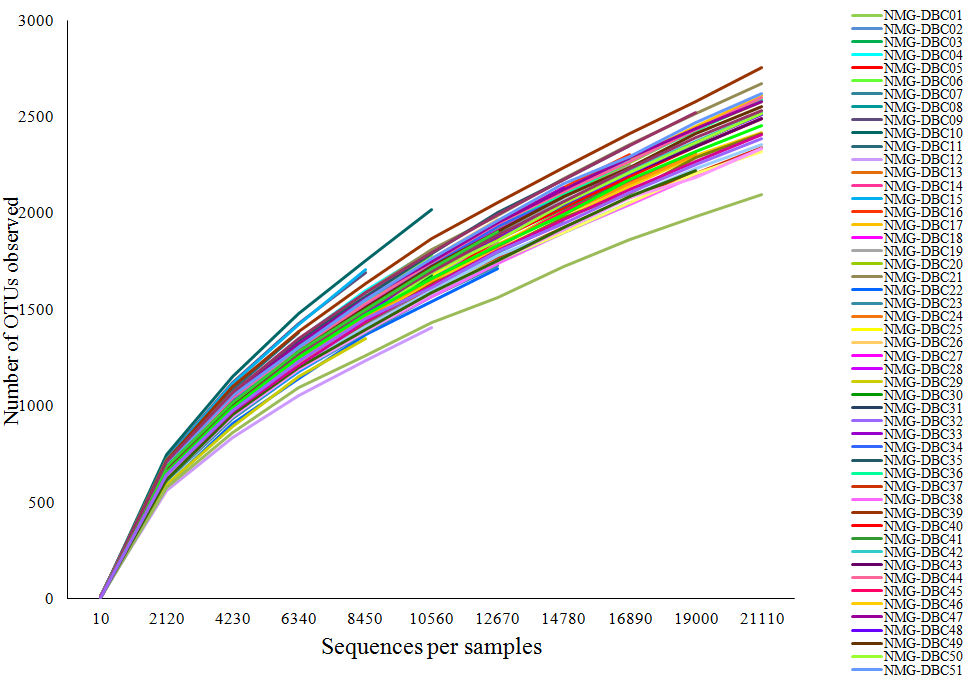


**S2 Fig. Rarefaction analysis of the IMG-DBC population group. Rarefaction curves of OTUs clustered at 97% sequence identity. (TIF)**
