# Supplementary material for: Comparative analysis of fecal microbial communities in cattle and Bactrian camels
Source: PLoS One. 2017 Mar 16;12(3):e0173062. doi: 10.1371/journal.pone.0173062 (PMC5354269; doi:10.1371/journal.pone.0173062)
Supplement: S1 Table — (DOC) [file pone.0173062.s005.doc]

**S1 Table. Number of OTUs and estimators of sequence diversity and richness. (DOC)**

| Population group | Sample  No. | OTUs  (97% identity) | Chao1 (Richness) | Shannon (Diversity) | Goods’s coverage |
| --- | --- | --- | --- | --- | --- |
| IMG-Cattle | 01 | 34133 | 7675 | 6.10 | 0.95 |
|  | 02 | 56193 | 12147 | 5.95 | 0.96 |
|  | 03 | 41948 | 8731 | 5.69 | 0.95 |
|  | 04 | 33366 | 7970 | 5.93 | 0.95 |
|  | 05 | 37368 | 9127 | 6.09 | 0.94 |
|  | 06 | 32091 | 8390 | 5.81 | 0.94 |
|  | 07 | 29019 | 7634 | 5.91 | 0.94 |
|  | 08 | 30897 | 8182 | 5.84 | 0.94 |
|  | 09 | 31084 | 9271 | 5.89 | 0.94 |
|  | 10 | 48277 | 9753 | 5.88 | 0.95 |
|  | 11 | 24085 | 6464 | 6.00 | 0.94 |
|  | 12 | 27500 | 6906 | 5.71 | 0.94 |
|  | 13 | 30667 | 7486 | 5.69 | 0.94 |
|  | 14 | 30046 | 8252 | 5.81 | 0.94 |
| IMG-DBC | 01 | 13414 | 4864 | 5.91 | 0.92 |
|  | 02 | 19152 | 6302 | 6.13 | 0.92 |
|  | 03 | 33694 | 7119 | 5.52 | 0.95 |
|  | 04 | 44449 | 8859 | 5.98 | 0.95 |
|  | 05 | 42100 | 9139 | 5.92 | 0.95 |
|  | 06 | 43862 | 9427 | 5.90 | 0.95 |
|  | 07 | 48461 | 10645 | 5.78 | 0.95 |
|  | 08 | 25269 | 7406 | 5.92 | 0.94 |
|  | 09 | 20608 | 5966 | 5.69 | 0.93 |
|  | 10 | 23790 | 6860 | 5.84 | 0.94 |
|  | 11 | 25776 | 6752 | 5.96 | 0.94 |
|  | 12 | 14662 | 5211 | 5.90 | 0.92 |
|  | 13 | 16679 | 5408 | 5.91 | 0.93 |
|  | 14 | 17729 | 5726 | 5.74 | 0.93 |
|  | 15 | 11957 | 4955 | 6.03 | 0.90 |
|  | 16 | 11029 | 4521 | 5.73 | 0.90 |
|  | 17 | 10701 | 4629 | 5.86 | 0.90 |
|  | 18 | 8080 | 3844 | 5.67 | 0.89 |
|  | 19 | 8279 | 3675 | 5.47 | 0.89 |
|  | 20 | 9586 | 4818 | 6.01 | 0.89 |
|  | 21 | 11114 | 5555 | 6.10 | 0.89 |
|  | 22 | 6859 | 3413 | 5.86 | 0.87 |
|  | 23 | 12051 | 3539 | 5.69 | 0.93 |
|  | 24 | 14733 | 5161 | 5.94 | 0.92 |
|  | 25 | 13598 | 4403 | 5.81 | 0.92 |
|  | 26 | 9028 | 4783 | 6.00 | 0.88 |
|  | 27 | 17284 | 5671 | 5.92 | 0.92 |
|  | 28 | 21301 | 6681 | 5.90 | 0.93 |
|  | 29 | 14808 | 4850 | 5.86 | 0.92 |
|  | 30 | 17061 | 5564 | 5.99 | 0.92 |
|  | 31 | 21542 | 6486 | 6.00 | 0.93 |
|  | 32 | 21386 | 7153 | 6.15 | 0.93 |
|  | 33 | 12820 | 4200 | 5.61 | 0.92 |
|  | 34 | 13840 | 4006 | 5.76 | 0.93 |
|  | 35 | 11302 | 4089 | 6.00 | 0.91 |
|  | 36 | 15587 | 5174 | 5.95 | 0.92 |
|  | 37 | 13232 | 4717 | 5.98 | 0.91 |
|  | 38 | 13085 | 4720 | 5.79 | 0.91 |
|  | 39 | 15558 | 5411 | 6.10 | 0.92 |
|  | 40 | 10141 | 3440 | 5.68 | 0.92 |
|  | 41 | 11889 | 4513 | 5.77 | 0.91 |
|  | 42 | 14350 | 6019 | 5.84 | 0.91 |
|  | 43 | 13201 | 4536 | 5.79 | 0.92 |
|  | 44 | 16564 | 5928 | 5.99 | 0.92 |
|  | 45 | 18470 | 6194 | 6.04 | 0.92 |
|  | 46 | 16939 | 6380 | 6.05 | 0.92 |
|  | 47 | 33461 | 9235 | 6.14 | 0.94 |
|  | 48 | 28252 | 7363 | 5.83 | 0.94 |
|  | 49 | 22750 | 6929 | 5.94 | 0.94 |
|  | 50 | 22622 | 7083 | 6.18 | 0.93 |
|  | 51 | 24471 | 7360 | 5.80 | 0.93 |
|  | 52 | 22565 | 6904 | 5.92 | 0.93 |
|  | 53 | 27223 | 7290 | 6.11 | 0.94 |
|  | 54 | 25490 | 6565 | 6.02 | 0.94 |
|  | 55 | 30959 | 9200 | 6.14 | 0.94 |
|  | 56 | 20922 | 5888 | 5.86 | 0.93 |
|  | 57 | 33588 | 8775 | 5.92 | 0.94 |
|  | 58 | 32041 | 8723 | 6.13 | 0.94 |
|  | 59 | 25559 | 7310 | 5.82 | 0.93 |
|  | 60 | 23831 | 7078 | 5.96 | 0.93 |
|  | 61 | 24669 | 7638 | 5.75 | 0.93 |
|  | 62 | 23318 | 7689 | 6.14 | 0.93 |
|  | 63 | 27573 | 8044 | 5.84 | 0.94 |
|  | 64 | 16959 | 4775 | 5.73 | 0.93 |
|  | 65 | 19795 | 5862 | 5.77 | 0.93 |
| MG-DBC | 01 | 15230 | 5433 | 5.35 | 0.92 |
|  | 02 | 13703 | 4431 | 4.75 | 0.94 |
|  | 03 | 15681 | 4609 | 5.63 | 0.93 |
| MG-WBC | 01 | 27649 | 6723 | 5.32 | 0.95 |
|  | 02 | 25467 | 6650 | 5.64 | 0.94 |
|  | 03 | 26947 | 7136 | 5.53 | 0.94 |
|  | 04 | 32924 | 7699 | 5.50 | 0.95 |
|  | 05 | 28070 | 7030 | 5.26 | 0.95 |
|  | 06 | 21996 | 6083 | 5.45 | 0.94 |
|  | 07 | 14902 | 5042 | 5.39 | 0.92 |
|  | 08 | 15963 | 5130 | 5.09 | 0.93 |
|  | 09 | 16064 | 5165 | 5.12 | 0.93 |
|  | 10 | 13709 | 4508 | 5.25 | 0.92 |
|  | 11 | 13990 | 5033 | 5.45 | 0.92 |
|  | 12 | 15948 | 4948 | 5.23 | 0.93 |
