# Supplementary material for: Comparative analysis of fecal microbial communities in cattle and Bactrian camels
Source: PLoS One. 2017 Mar 16;12(3):e0173062. doi: 10.1371/journal.pone.0173062 (PMC5354269; doi:10.1371/journal.pone.0173062)
Supplement: S2 Table — (DOC) [file pone.0173062.s006.doc]

**S2 Table. Detailed Taxonomic string for the 47 significant different OTUs between the MG-WBC and IMG-DBC groups (classified to genus, p<0.05. Control-IMG-DBC). (DOC)**

| Clustered ID | t-test | fold-change | Consensus lineage |
| --- | --- | --- | --- |
| 68 | 4.4216E-16 | -2.453575189 | Bacteria;Firmicutes;Clostridia;Clostridiales;Lachnospiraceae;Pseudobutyrivibrio |
| 61 | 6.40436E-16 | -2.187800761 | Bacteria;Firmicutes;Erysipelotrichi;Erysipelotrichales;Erysipelotrichaceae;Coprobacillus |
| 30 | 7.86149E-14 | -2.306587298 | Bacteria;Firmicutes;Clostridia;Clostridiales;Lachnospiraceae;Butyrivibrio |
| 33 | 6.71901E-12 | 1.437761071 | Bacteria;Firmicutes;Clostridia;Clostridiales;Ruminococcaceae;Sporobacter |
| 79 | 3.03001E-11 | -1.248827617 | Bacteria;Tenericutes;Mollicutes;Anaeroplasmatales;Anaeroplasmataceae;Anaeroplasma |
| 63 | 1.50755E-09 | -1.840993349 | Bacteria;Proteobacteria;Gammaproteobacteria;Aeromonadales;Succinivibrionaceae;Succinivibrio |
| 77 | 2.1304E-08 | 1.637341615 | Bacteria;Firmicutes;Clostridia;Clostridiales;Eubacteriaceae;Anaerofustis |
| 80 | 4.8851E-08 | -1.526134343 | Bacteria;Verrucomicrobia;Opitutae;Puniceicoccales;Puniceicoccaceae;Cerasicoccus |
| 65 | 1.79985E-07 | -1.952111625 | Archaea;Euryarchaeota;Methanomicrobia;Methanomicrobiales;Methanocorpusculaceae;Methanocorpusculum |
| 35 | 1.96191E-07 | 1.278799683 | Bacteria;Firmicutes;Clostridia;Clostridiales;Lachnospiraceae;Syntrophococcus |
| 108 | 4.31106E-07 | 1.342372931 | Bacteria;Actinobacteria;Actinobacteria;Coriobacteriales;Coriobacteriaceae;Olsenella |
| 10 | 8.37102E-07 | -2.101950937 | Bacteria;Lentisphaerae;Lentisphaeria;Victivallales;Victivallaceae;Victivallis |
| 153 | 3.964E-06 | -1.114416746 | Bacteria;Firmicutes;Clostridia;Clostridiales;Veillonellaceae;Schwartzia |
| 157 | 1.02291E-05 | 1.411630705 | Bacteria;Firmicutes;Bacilli;Bacillales;Bacillaceae;Lysinibacillus |
| 99 | 4.23164E-05 | 1.857572297 | Bacteria;Proteobacteria;Epsilonproteobacteria;Campylobacterales;Helicobacteraceae;Helicobacter |
| 140 | 6.42899E-05 | -1.163779024 | Bacteria;Firmicutes;Clostridia;Clostridiales;Veillonellaceae;Selenomonas |
| 106 | 0.000103549 | -1.240641336 | Bacteria;Bacteroidetes;Bacteroidia;Bacteroidales;Porphyromonadaceae;Paludibacter |
| 83 | 0.000325972 | -1.472750547 | Bacteria;Fibrobacteres;Fibrobacteria;Fibrobacterales;Fibrobacteraceae;Fibrobacter |
| 66 | 0.000379824 | 1.236661044 | Bacteria;Firmicutes;Clostridia;Clostridiales;Lachnospiraceae;Dorea |
| 49 | 0.000404388 | 1.579271545 | Bacteria;Firmicutes;Bacilli;Bacillales;Planococcaceae;Kurthia |
| 52 | 0.000690675 | -1.206119785 | Archaea;Euryarchaeota;Methanobacteria;Methanobacteriales;Methanobacteriaceae;Methanosphaera |
| 88 | 0.001059394 | 1.291221033 | Bacteria;Firmicutes;Clostridia;Clostridiales;Ruminococcaceae;Butyricicoccus |
| 227 | 0.001303757 | -1.070441377 | Bacteria;Firmicutes;Bacilli;Lactobacillales;Enterococcaceae;Enterococcus |
| 103 | 0.001494977 | -1.065507345 | Bacteria;Firmicutes;Clostridia;Clostridiales;Lachnospiraceae;Anaerosporobacter |
| 208 | 0.001799839 | 1.160302625 | Bacteria;Proteobacteria;Gammaproteobacteria;Pseudomonadales;Moraxellaceae;Acinetobacter |
| 178 | 0.002095683 | -1.134951885 | Bacteria;Fusobacteria;Fusobacteria;Fusobacteriales;Fusobacteriaceae;Fusobacterium |
| 53 | 0.002468241 | -1.277595997 | Bacteria;Firmicutes;Bacilli;Lactobacillales;Streptococcaceae;Streptococcus |
| 16 | 0.00456475 | 1.105101377 | Bacteria;Firmicutes;Clostridia;Clostridiales;Ruminococcaceae;Oscillibacter |
| 207 | 0.005277898 | -1.047251896 | Bacteria;Proteobacteria;Epsilonproteobacteria;Campylobacterales;Helicobacteraceae;Wolinella |
| 25 | 0.007394153 | 1.225631368 | Bacteria;Bacteroidetes;Bacteroidia;Bacteroidales;Rikenellaceae;Alistipes |
| 100 | 0.012495196 | -1.198741117 | Archaea;Euryarchaeota;Methanobacteria;Methanobacteriales;Methanobacteriaceae;Methanobrevibacter |
| 55 | 0.014857384 | -1.144700986 | Bacteria;Firmicutes;Clostridia;Clostridiales;Ruminococcaceae;Papillibacter |
| 130 | 0.016660709 | -1.261830134 | Bacteria;Firmicutes;Clostridia;Clostridiales;Veillonellaceae;Anaerovibrio |
| 238 | 0.025403429 | 1.013473668 | Bacteria;Actinobacteria;Actinobacteria;Actinomycetales;Mycobacteriaceae;Mycobacterium |
| 50 | 0.028521157 | 1.165688389 | Bacteria;Firmicutes;Clostridia;Clostridiales;Ruminococcaceae;Acetivibrio |
| 84 | 0.031596658 | 1.013470127 | Bacteria;Actinobacteria;Actinobacteria;Actinomycetales;Nocardioidaceae;Marmoricola |
| 97 | 0.032793417 | -1.161257615 | Bacteria;Proteobacteria;Gammaproteobacteria;Aeromonadales;Succinivibrionaceae;Ruminobacter |
| 247 | 0.034029743 | 1.013395118 | Bacteria;Proteobacteria;Gammaproteobacteria;Alteromonadales;Alteromonadaceae;Glaciecola |
| 112 | 0.035776595 | 1.013307651 | Bacteria;Firmicutes;Bacilli;Bacillales;Bacillaceae;Geobacillus |
| 164 | 0.035776595 | 1.013307651 | Bacteria;Bacteroidetes;Sphingobacteria;Sphingobacteriales;Chitinophagaceae;Flavisolibacter |
| 200 | 0.035776595 | 1.013307651 | Bacteria;Proteobacteria;Gammaproteobacteria;Aeromonadales;Succinivibrionaceae;Succinimonas |
| 205 | 0.036447194 | 1.095152056 | Bacteria;Actinobacteria;Actinobacteria;Actinomycetales;Cellulomonadaceae;Cellulomonas |
| 111 | 0.038652143 | 1.073606957 | Bacteria;Actinobacteria;Actinobacteria;Coriobacteriales;Coriobacteriaceae;Slackia |
| 198 | 0.041495758 | 1.013294545 | Bacteria;Firmicutes;Bacilli;Lactobacillales;Carnobacteriaceae;Atopostipes |
| 248 | 0.041848317 | 1.013128758 | Bacteria;Proteobacteria;Betaproteobacteria;Burkholderiales;Burkholderiaceae;Chitinimonas |
| 138 | 0.043617193 | -1.176347008 | Bacteria;Firmicutes;Clostridia;Clostridiales;Incertae Sedis XIII;Mogibacterium |
| 47 | 0.045714061 | 1.01324432 | Bacteria;Bacteroidetes;Flavobacteria;Flavobacteriales;Flavobacteriaceae;Cloacibacterium |
