# Supplementary material for: Comparative analysis of fecal microbial communities in cattle and Bactrian camels
Source: PLoS One. 2017 Mar 16;12(3):e0173062. doi: 10.1371/journal.pone.0173062 (PMC5354269; doi:10.1371/journal.pone.0173062)
Supplement: S3 Table — (DOC) [file pone.0173062.s007.doc]

**S3 Table. Detailed Taxonomic string for the 48 significant different OTUs between the IMG-DBC and IMG-Cattle groups (classified to genus, p<0.05. Control-IMG-Cattle). (DOC)**

| Clustered ID | t-test | fold-change | Consensus lineage |
| --- | --- | --- | --- |
| 148 | 8.43255E-30 | 4.187598958 | Bacteria;Proteobacteria;Deltaproteobacteria;Desulfovibrionales;Desulfovibrionaceae;Desulfovibrio |
| 33 | 6.85022E-17 | -1.569774574 | Bacteria;Firmicutes;Clostridia;Clostridiales;Ruminococcaceae;Sporobacter |
| 68 | 1.22564E-16 | 2.338265013 | Bacteria;Firmicutes;Clostridia;Clostridiales;Lachnospiraceae;Pseudobutyrivibrio |
| 72 | 9.9319E-15 | -2.614019652 | Bacteria;Firmicutes;Clostridia;Clostridiales;Clostridiaceae;Clostridium |
| 130 | 8.77161E-14 | 1.458510987 | Bacteria;Firmicutes;Clostridia;Clostridiales;Veillonellaceae;Anaerovibrio |
| 30 | 7.51889E-13 | 2.231825717 | Bacteria;Firmicutes;Clostridia;Clostridiales;Lachnospiraceae;Butyrivibrio |
| 97 | 2.43016E-12 | 1.285872319 | Bacteria;Proteobacteria;Gammaproteobacteria;Aeromonadales;Succinivibrionaceae;Ruminobacter |
| 63 | 2.61394E-11 | 1.910751824 | Bacteria;Proteobacteria;Gammaproteobacteria;Aeromonadales;Succinivibrionaceae;Succinivibrio |
| 10 | 7.53421E-11 | 1.339757384 | Bacteria;Lentisphaerae;Lentisphaeria;Victivallales;Victivallaceae;Victivallis |
| 99 | 9.40143E-11 | 1.563496879 | Bacteria;Proteobacteria;Epsilonproteobacteria;Campylobacterales;Helicobacteraceae;Helicobacter |
| 66 | 7.32131E-10 | -1.442265536 | Bacteria;Firmicutes;Clostridia;Clostridiales;Lachnospiraceae;Dorea |
| 140 | 1.49523E-09 | 1.207762838 | Bacteria;Firmicutes;Clostridia;Clostridiales;Veillonellaceae;Selenomonas |
| 88 | 1.57091E-09 | 1.646200282 | Bacteria;Firmicutes;Clostridia;Clostridiales;Ruminococcaceae;Butyricicoccus |
| 60 | 3.76601E-08 | -1.438253179 | Bacteria;Spirochaetes;Spirochaetes;Spirochaetales;Spirochaetaceae;Treponema |
| 79 | 4.43469E-08 | 1.225779512 | Bacteria;Tenericutes;Mollicutes;Anaeroplasmatales;Anaeroplasmataceae;Anaeroplasma |
| 77 | 1.96676E-07 | -1.544741339 | Bacteria;Firmicutes;Clostridia;Clostridiales;Eubacteriaceae;Anaerofustis |
| 42 | 2.95753E-07 | -1.417222121 | Bacteria;Firmicutes;Erysipelotrichi;Erysipelotrichales;Erysipelotrichaceae;Holdemania |
| 153 | 1.17884E-06 | 1.130775762 | Bacteria;Firmicutes;Clostridia;Clostridiales;Veillonellaceae;Schwartzia |
| 100 | 1.93497E-06 | -1.744394919 | Archaea;Euryarchaeota;Methanobacteria;Methanobacteriales;Methanobacteriaceae;Methanobrevibacter |
| 16 | 3.88299E-06 | -1.173938261 | Bacteria;Firmicutes;Clostridia;Clostridiales;Ruminococcaceae;Oscillibacter |
| 135 | 4.09288E-06 | 1.192357068 | Bacteria;Actinobacteria;Actinobacteria;Coriobacteriales;Coriobacteriaceae;Enterorhabdus |
| 85 | 6.09746E-06 | 1.141602435 | Bacteria;Firmicutes;Erysipelotrichi;Erysipelotrichales;Erysipelotrichaceae;Bulleidia |
| 109 | 6.25963E-06 | 1.453881784 | Bacteria;Bacteroidetes;Bacteroidia;Bacteroidales;Porphyromonadaceae;Parabacteroides |
| 25 | 6.76685E-06 | -1.394253094 | Bacteria;Bacteroidetes;Bacteroidia;Bacteroidales;Rikenellaceae;Alistipes |
| 55 | 9.86428E-06 | -1.286719438 | Bacteria;Firmicutes;Clostridia;Clostridiales;Ruminococcaceae;Papillibacter |
| 23 | 2.25367E-05 | -1.287184669 | Bacteria;Firmicutes;Clostridia;Clostridiales;Lachnospiraceae;Coprococcus |
| 61 | 2.45437E-05 | 1.478293381 | Bacteria;Firmicutes;Erysipelotrichi;Erysipelotrichales;Erysipelotrichaceae;Coprobacillus |
| 218 | 0.000121234 | -1.224986572 | Bacteria;Deferribacteres;Deferribacteres;Deferribacterales;Deferribacteraceae;Mucispirillum |
| 62 | 0.000122521 | -1.358747861 | Bacteria;Firmicutes;Clostridia;Clostridiales;Ruminococcaceae;Faecalibacterium |
| 227 | 0.000381732 | 1.085459221 | Bacteria;Firmicutes;Bacilli;Lactobacillales;Enterococcaceae;Enterococcus |
| 54 | 0.000793281 | -1.334246513 | Bacteria;Firmicutes;Erysipelotrichi;Erysipelotrichales;Erysipelotrichaceae;Turicibacter |
| 14 | 0.001107543 | 1.178688681 | Bacteria;Firmicutes;Clostridia;Clostridiales;Incertae Sedis XIV;Blautia |
| 104 | 0.001231506 | 1.085864288 | Bacteria;Firmicutes;Bacilli;Lactobacillales;Streptococcaceae;Lactococcus |
| 207 | 0.001469093 | 1.061586257 | Bacteria;Proteobacteria;Epsilonproteobacteria;Campylobacterales;Helicobacteraceae;Wolinella |
| 116 | 0.004724002 | 1.106221429 | Bacteria;Proteobacteria;Epsilonproteobacteria;Campylobacterales;Campylobacteraceae;Campylobacter |
| 178 | 0.004993629 | 1.132578079 | Bacteria;Fusobacteria;Fusobacteria;Fusobacteriales;Fusobacteriaceae;Fusobacterium |
| 18 | 0.005633118 | -1.155254269 | Bacteria;Firmicutes;Clostridia;Clostridiales;Lachnospiraceae;Roseburia |
| 35 | 0.006717416 | -1.250659215 | Bacteria;Firmicutes;Clostridia;Clostridiales;Lachnospiraceae;Syntrophococcus |
| 118 | 0.01123677 | 1.110995352 | Bacteria;Firmicutes;Clostridia;Clostridiales;Veillonellaceae;Succiniclasticum |
| 208 | 0.011845524 | -1.195394411 | Bacteria;Proteobacteria;Gammaproteobacteria;Pseudomonadales;Moraxellaceae;Acinetobacter |
| 22 | 0.012977223 | -1.080571178 | Bacteria;Firmicutes;Clostridia;Clostridiales;Gracilibacteraceae;Lutispora |
| 103 | 0.017421045 | 1.062918649 | Bacteria;Firmicutes;Clostridia;Clostridiales;Lachnospiraceae;Anaerosporobacter |
| 189 | 0.021555891 | -1.214033639 | Bacteria;Proteobacteria;Gammaproteobacteria;Pseudomonadales;Pseudomonadaceae;Pseudomonas |
| 12 | 0.029804115 | -1.158803609 | Bacteria;Firmicutes;Clostridia;Clostridiales;Ruminococcaceae;Ruminococcus |
| 52 | 0.033388877 | -1.174670281 | Archaea;Euryarchaeota;Methanobacteria;Methanobacteriales;Methanobacteriaceae;Methanosphaera |
| 221 | 0.042484151 | -1.071342776 | Bacteria;Actinobacteria;Actinobacteria;Actinomycetales;Yaniellaceae;Yaniella |
| 83 | 0.043456019 | 1.488358181 | Bacteria;Fibrobacteres;Fibrobacteria;Fibrobacterales;Fibrobacteraceae;Fibrobacter |
| 230 | 0.046061932 | -1.03997702 | Bacteria;Actinobacteria;Actinobacteria;Actinomycetales;Nocardioidaceae;Aeromicrobium |
